# Simvastatin rescues memory and granule cell maturation through the Wnt/β-catenin signaling pathway in a mouse model of Alzheimer’s disease

**DOI:** 10.1038/s41419-022-04784-y

**Published:** 2022-04-09

**Authors:** Xin-Kang Tong, Jessika Royea, Edith Hamel

**Affiliations:** 1 Laboratory of Cerebrovascular Research, Montreal Neurological Institute, McGill University, 3801 University Street, H3A 2B4 Montréal, QC Canada; 2 Present Address: Department of Biochemistry, Microbiology, Immunology University of Ottawa, Ottawa, ON K1H 8M5 Canada

**Keywords:** Alzheimer's disease, Adult neurogenesis

## Abstract

We previously showed that simvastatin (SV) restored memory in a mouse model of Alzheimer disease (AD) concomitantly with normalization in protein levels of memory-related immediate early genes in hippocampal CA1 neurons. Here, we investigated age-related changes in the hippocampal memory pathway, and whether the beneficial effects of SV could be related to enhanced neurogenesis and signaling in the Wnt/β-catenin pathway. APP mice and wild-type (WT) littermate controls showed comparable number of proliferating (Ki67-positive nuclei) and immature (doublecortin (DCX)-positive) granule cells in the dentate gyrus until 3 months of age. At 4 months, Ki67 or DCX positive cells decreased sharply and remained less numerous until the endpoint (6 months) in both SV-treated and untreated APP mice. In 6 month-old APP mice, dendritic extensions of DCX immature neurons in the molecular layer were shorter, a deficit fully normalized by SV. Similarly, whereas mature granule cells (calbindin-immunopositive) were decreased in APP mice and not restored by SV, their dendritic arborizations were normalized to control levels by SV treatment. SV increased Prox1 protein levels (↑67.7%, *p* < 0.01), a Wnt/β-catenin signaling target, while significantly decreasing (↓61.2%, *p* < 0.05) the upregulated levels of the β-catenin-dependent Wnt pathway inhibitor DKK1 seen in APP mice. In APP mice, SV benefits were recapitulated by treatment with the Wnt/β-catenin specific agonist WAY-262611, whereas they were fully abolished in mice that received the Wnt/β-catenin pathway inhibitor XAV939 during the last month of SV treatment. Our results indicate that activation of the Wnt-β-catenin pathway through downregulation of DKK1 underlies SV neuronal and cognitive benefits.

## Introduction

Alzheimer’s disease (AD) is the leading cause of dementia worldwide and despite its deleterious personal, familial, and societal effects, we have little to offer to its growing patient population. Recently, cardiovascular diseases have been identified as the main risk factor for the late onset, non-genetic, form of AD that represents the bulk of AD cases. For this reason, emphasis has been put on the treatment of cardiovascular diseases as a promising approach to delay or counter AD manifestations [1]. In this regard, repurposing drugs already available for treating hypercholesterolemia, such as statins, has been highlighted as a means to reduce the incidence of AD, slow down its progression, and lighten the burden of this devastating neurodegenerative disease [2]. Indeed, epidemiological studies and meta-analyses have reported that statins, particularly those that cross the blood-brain-barrier and access the brain parenchyma like simvastatin (SV), decrease the risk of AD and dementia and improve cognitive performance in AD patients [3–5]. Most recent meta-analyses [4, 6–9] revealed that statins are associated with a decreased risk for AD in both males and females, with a potentially greater efficacy for patients homozygous for ApoE4 [10] and those treated with high-potency statins [11]. While these benefits may be drug, sex- and race-ethnicity related [12], well-controlled clinical trials are still needed on large at-risk populations to validate the epidemiological and data mining studies. Nevertheless, several pre-clinical studies using statins in animal models of AD strongly support their potential therapeutic value. Particularly relevant are reports of improved or fully restored memory, hippocampal and cerebrovascular function in various AD animal models [13–19]. These benefits occurred without reducing the classic AD biomarker, amyloid-β (Aβ) levels or Aβ plaque load [14, 15, 20, 21], emphasizing the need to explore therapeutic avenues directed at targets other than the Aβ pathology, as supported by the repeated failure of Aβ-directed approaches throughout the years [22, 23].

In this respect, independent from their intended cholesterol lowering effects, statins exert multiple antioxidative, anti-inflammatory, and neuroprotective pleiotropic benefits [24]. We previously found that SV rescued memory in adult transgenic AD mice that overexpress a mutated form of the human amyloid precursor protein (APP mice) together with normalized or upregulated protein levels of learning- and memory-related immediate early genes c-Fos or Egr-1 in hippocampal CA1 neurons, with no effect on Aβ load, synaptic proteins or NMDA receptor subunits [15]. Recently, activation of the Wnt/β-catenin pathway has been linked to statins anti-inflammatory and neuroprotective effects [25–27], and suggested as a new pharmacological target for statins [28]. This is highly relevant to AD since deficiency of the canonical Wnt/β-catenin signaling pathway has been proposed as a triggering pathogenic factor [29]. In the present study, we explored the age-related cellular alterations in the dentate gyrus (DG) granule cells of APP mice and further attempted to decipher the role of the Wnt signaling pathway in SV’s protective effects on memory, as well as the hippocampal memory circuit and structure in adult APP mice.

## Results

Data are available from the corresponding author upon reasonable request.

### SV did not affect the number of proliferating cells or immature neurons in the DG area

**Fig. 1:**
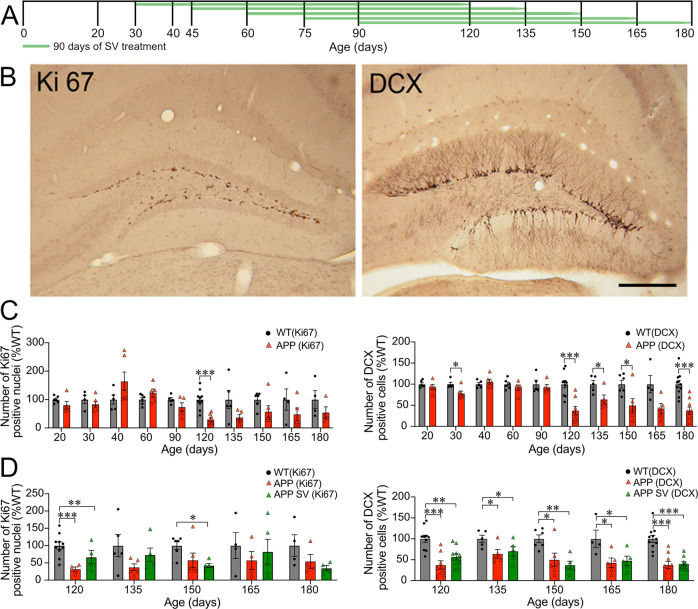
Adult APP mice have decreased cell proliferation and immature neurons in the dentate gyrus. **A** Schematic representation of experimental design and timeline of SV treatment. **B** Proliferative Ki67-immunopositive nuclei and doublecortin (DCX)-immunostained cell bodies and dendritic projections visualized with DAB in the subgranular zone of the dentate gyrus (DG). **C** Ki67-positive nuclei and immature DCX-positive neurons were comparable in number between APP and WT until 120 days of age. As of 120 days, a significant decrease in the number of both populations was observed that persisted until 180 days of age, being significant at all time points for DCX neurons. **D** SV treatment (90 days) did not affect the number of Ki67 and DCX positive neurons irrespective of the age of treatment initiation as reduced numbers of both cell populations compared to WT were found at all endpoints (120-180 days), being significant for DCX neurons at all ages. Cell nuclei (Ki67) and cell bodies (DCX) were counted in tissue sections from APP and WT mice (4–6 mice/group), and expressed as percent of WT (dotted line) at different ages (20 to 180 days). Scale bar = 200 µm. *p < 0.05, **p < 0.01, and ***p < 0.001, using multiple repeated Student’s *t* tests (**C**) or one-way ANOVAs (**D**).

Between 20 to 180 days of age (Fig. 1A), cell proliferation and maturation in the granule cell layer of the DG was assessed with Ki67 and DCX, respectively, and different patterns were observed between APP and WT mice. In young APP mice (20–30 days old), the number of both proliferating cells with Ki67-immunopositive nuclei and migrating immature DCX-positive neurons (Fig. 1B) was slightly less (~20%, ns or *p* < 0.05 at 30 days for DCX) than WT mice (Fig. 1C). Ki67-immunostained nuclei increased in number in APP mice by 40 days of age (>50% more than in WT mice), which was followed by a progressive decline until 120 days (Fig. 1C, *p* < 0.001) whereby a plateau was reached corresponding to ~50% of WT Ki67 stained nuclei. In contrast, immature DCX granule cells were relatively stable in APP mice and compared well to WT in number until 90 days of age. They abruptly decreased by 120 days (Fig. 1C, *p* < 0.001) and remained stable thereafter corresponding to ~30–60% of WT DCX neurons. When comparing APP mice treated or not with SV for 90 days, irrespective of the age at which treatment was initiated (30, 45, 60, 75, or 90 days) (Fig. 1A), the number of DG proliferating (Ki67-positive nuclei) and immature (DCX-positive cells) neurons was less (~50% through age) compared to WT mice (Fig. 1D), and there was no statistical difference between APP and SV-treated APP mice in the number of Ki67 and DCX cells in all age groups (Fig. 1D).

### SV restored dendritic extensions of immature granule cells in the DG molecular layer

**Fig. 2:**
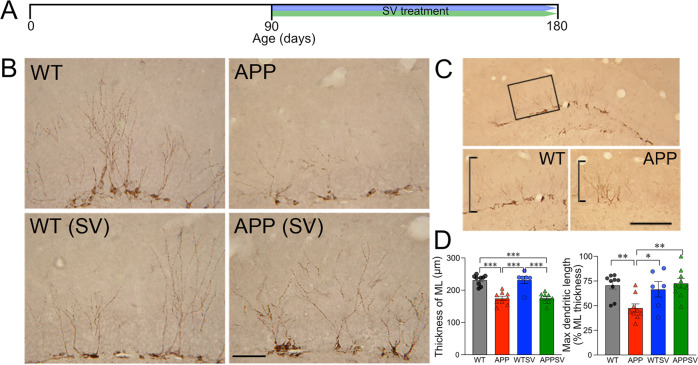
SV normalized dendritic outgrowth of DCX immature neurons in APP mice. **A** Schematic representation of experimental design and timeline of SV treatment. **B** DCX-positive immature neurons were visualized by DAB immunohistochemistry in the DG of WT, APP, and WT and APP mice treated with SV (WTSV and APPSV). **C** The black box in the top panel indicates the target area for measuring the thickness of the molecular layer (ML) of the DG. The ML in APP mice was statistically thinner than that of WT mice, and there was no difference between treated and untreated animals (**D** left panel). After correcting for the reduced thickness of the ML, the maximum length of DCX-immunostained dendritic extensions was shorter in APP mice compared to WT (**D** right panel), a deficit that was fully corrected by SV treatment that promoted extension of the DCX dendrites in treated APP mice to levels comparable to WT (**D** right panel). Scale bars = 200 µm. *p < 0.05, **p < 0.01, and ***p < 0.001, using two-way ANOVA followed by Newman–Keuls post-hoc test.

In adult (180 days old, Fig. 2A) APP mice, remaining DCX-immature neurons in the granule cell layer had shorter dendritic arborizations in the molecular layer of the DG compared to WT (Fig. 2B). After adjusting for a thinner molecular layer in APP mice, a structural change corresponding to ~25% reduction compared to WT (Fig. 2C, D left panel, *p* < 0.001), the reduced dendritic length of DCX neurons in the molecular layer was still observed with dendrites from APP mice extending less than 50% across the layer compared to ~75% in WT mice (Fig. 2D, right panel, *p* < 0.01). SV treatment (90 days, Fig. 2A) did not restore the molecular layer thickness, but fully normalized the dendritic length of remaining DCX cells that now extended up to ~75% across the molecular layer, as in WT mice (Fig. 2B bottom panels, D right panel).

### SV restored dendritic extensions of mature granule cells in the DG molecular layer

**Fig. 3:**
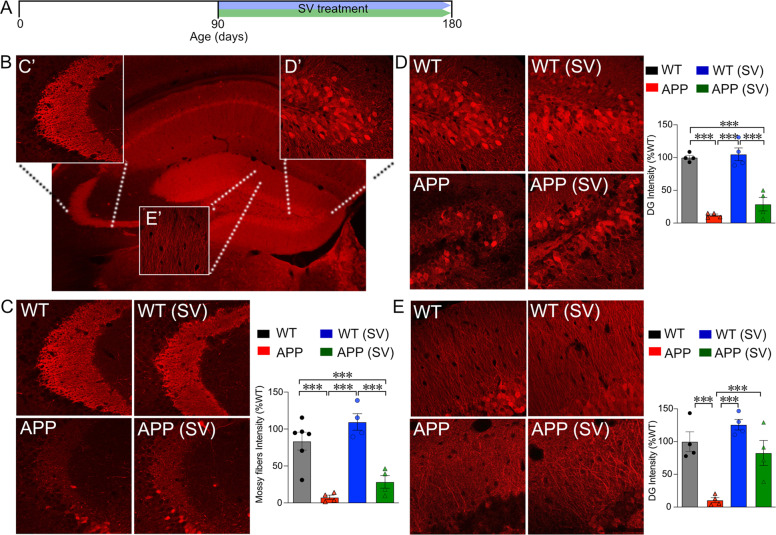
SV restored dendritic extensions of calbindin-immunolabelled mature granule cells in the dentate gyrus molecular layer in APP mice. **A** Schematic representation of experimental design and timeline of SV treatment. **B** A confocal image of calbindin-immunostained mature granule cells with their axonal projections through the mossy fibers in the CA3 area (C’), cell bodies at the level of the dentate gyrus apex area (DG, D’), and their dendritic arborizations in the molecular layer (E’). Quantitative analysis revealed dramatic decreases in the intensity of calbindin-immunostained mossy fibers (**C**) and cell bodies (**D**) in APP mice compared to WT controls, deficits that were not improved by SV treatment. In contrast, the drastic decrease in calbindin-immunostained dendritic extensions in the DG molecular layer was normalized to control levels in APP mice treated with SV (**E**). ***p < 0.001, using two-way ANOVA followed by Newman–Keuls post-hoc test.

Whereas DCX labels immature neurons in the granule cell layer of the DG, calbindin is a marker of mature cells and allows investigating putative changes in mature granule cells, their dendritic extensions in the molecular layer, and their axonal projections to the CA3 area through mossy fibers. In the CA3 region of the hippocampus (Fig. 3B, C’), the intensity of calbindin immunostained axonal afferents of the performant path and mossy fibers was drastically reduced (>80%, *p* < 0.001) in adult (180 days) APP mice compared to WT (Fig. 3C). SV treatment (Fig. 3A) increased calbindin fiber density in treated APP mice but did not correct this deficit (Fig. 3C). Similarly, the loss of calbindin-immunostained granule cell bodies (Fig. 3B, D’) in APP mice, although improved by SV treatment, was not recovered in APPSV mice compared to WT controls (Fig. 3D, *p* < 0.001). However, akin to the beneficial effect of SV on DCX-labeled dendritic extensions in the molecular layer, the reduced calbindin-immunolabeled dendritic projections (Fig. 3B, E’) in APP mice were normalized to WT levels by SV (Fig. 3E, ↑ 86.5%, *p* < 0.001).

### DKK1 affects cognitive function, β-catenin levels and dendritic length in APP mice

**Fig. 4:**
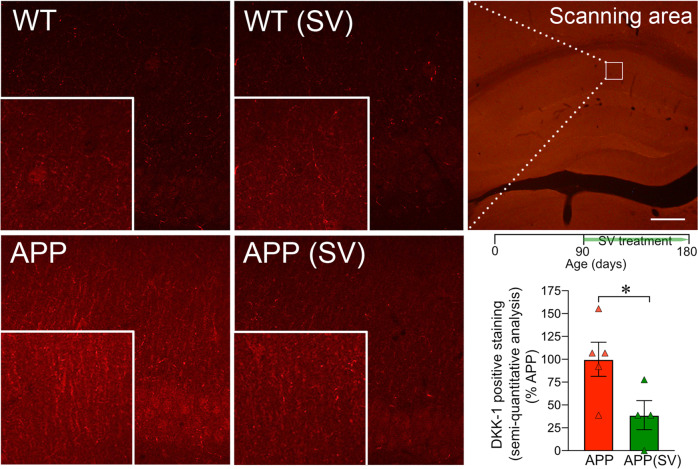
SV decreased DKK1 protein expression in the dentate gyrus of APP mice. Representative confocal scanning images show DKK1 immunopositive material in the dentate gyrus of WT and APP mice. The empty white box in the top right panel shows the scanning area illustrated in the other panels and used for analysis. Inserts at the bottom of each image indicate higher magnification of the same field. Semi-quantitative analysis performed in APP mice showed that SV (treatment timeline shown on top of bar graph) significantly decreased DKK1 protein expression in the CA1 stratum lacunosum-moleculare area compared to untreated APP. Scale bar = 45 µm. *p < 0.05, using Student’s *t* test.

The benefits of SV in the hippocampal memory pathway, namely restoration of dendritic arborizations of DG granule cells that receive afferents from the performant path, prompted us to investigate the role of the Wnt/β-catenin signaling pathway. Indeed, this pathway plays a crucial role in the development and maintenance of the hippocampus. DKK1, an endogenous protein acting as a selective antagonist of Wnt/β-catenin signaling and involved in memory [30], has been shown to be expressed at very low levels in the brain of young adults and elevated in that of AD patients [31] and AD mouse models [32]. Interestingly, in the hippocampus of APP mice, we found DKK1-immunopositive material appearing as small neurites predominantly visible in the CA1 stratum lacunosum-moleculare, a region important for memory formation since it receives performant path projections from the entorhinal cortex. In contrast, WT mice were literally devoid of such DKK1-immunopositive labeling (Fig. 4). Correspondingly, DKK1-positive material was severely reduced (↓62%, *p* < 0.05) in APPSV treated mice compared to untreated APP mice (Fig. 4), suggesting that SV likely mediates some of its benefits by reducing DKK1 expression.

**Fig. 5:**
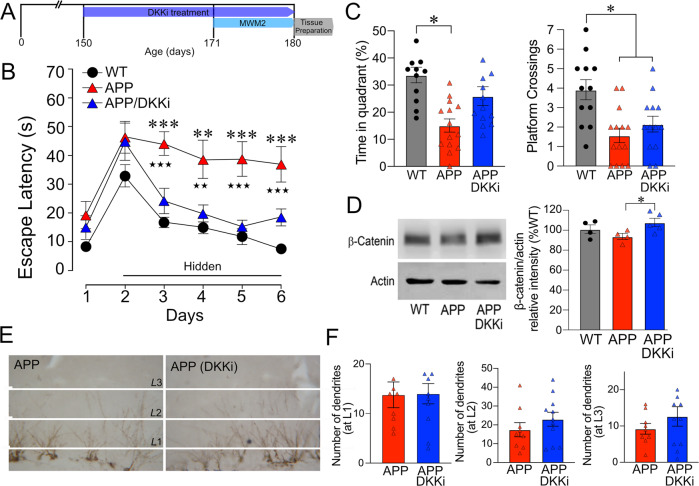
DKK1 inhibitor improved spatial learning and memory and hippocampal β-catenin protein levels in APP mice. **A** Schematic representation of experimental design and timeline of DKK1 inhibitor (DKKi) treatment. **B** One month of icv administration of the DKKi WAY-262611 restored spatial learning during all days of hidden platform testing in treated APP mice (blue triangle) compared to untreated APP mice that were severely impaired (red triangle) in the Morris water maze (MWM2) compared to both WT and APP mice treated with the DKKi. **C** During the probe trial, APP mice demonstrated deficits compared to WT, as shown here by a shorter time spent in the target quadrant and less crossings over the previous location of the platform. DKKi-treated APP mice had restored memory as shown by the same time spent in the target quadrant as WT controls, but precision was not improved as crossings over the previous location of the platform were still impaired. **D** Representative Western blots of β-catenin expression in WT, APP and DKKi-treated APP mice. Although hippocampal β-catenin protein levels were not significantly decreased in APP mice compared to WT, the DKKi WAY-262611 significantly increased them in treated APP mice. (**E**) DCX-immunostained dendritic extensions were counted in three segments (L1, L2, and L3) of the DG molecular layer in APP and DKKi-treated APP mice. Although more extensions were present in L2 and L3, it did not reach significance (**F**). *APP compared to WT (**B**); DKKi-treated APP compared to APP (**B**). *p < 0.05; **p < 0.01; ***p < 0.001 using one-way ANOVA and repeated measures ANOVA followed by Newman–Keuls post-hoc test.

We further tested whether DKK1 could affect cognitive function, β-catenin expression and granule cell dendritic outgrowth in the molecular layer of the DG (Fig. 5A). In the learning phase of the MWM, in agreement with our previous studies [14, 15], adult APP mice performed significantly worse than WT mice as shown by longer time latencies to find the hidden platform (Fig. 5B). Treatment with the selective activator of the Wnt/β-catenin pathway and DKK1 inhibitor (DKKi) WAY-262611 (icv, up to 30 days, Fig. 5A) significantly improved learning in APP mice in the hidden platform testing, DKKi-treated APP mice performing as well as WT mice (Fig. 5B). During the probe trial, APP mice showed deficits compared to WT, with shorter time spent in the target quadrant and less crossings over the previous location of the hidden platform (Fig. 5C). In contrast, DKKi-treated APP mice spent a comparable time to WT controls in the target quadrant where the hidden platform was previously located, although the number of crossings over the exact location of the platform was not improved (Fig. 5C). Protein levels of β-catenin were slightly reduced, albeit not significantly, in APP mice compared to WT controls, whereas β-catenin levels in DKKi-treated APP mice were significantly increased (Fig. 5D, *p* < 0.05, see Supplementary Fig. 1 for original blots) and fully normalized to WT levels. When investigating the number of DCX-immunopositive dendrites extending throughout the DG molecular layer (divided in L1-L3 segments, Fig. 5E) in APP and DKKi-treated APP mice, it was similar at L1, and slightly increased, albeit not significantly, at L2 and L3 in DKKi-treated APP mice (Fig. 5F). These results in APP mice indicate a deleterious role for high DKK1 levels on memory, Wnt/β-catenin signaling and, to some extent, on dendritic outgrowth of immature granule cells.

### β-catenin and Prox1 protein levels in the DG of APP mice: effects of SV

**Fig. 6:**
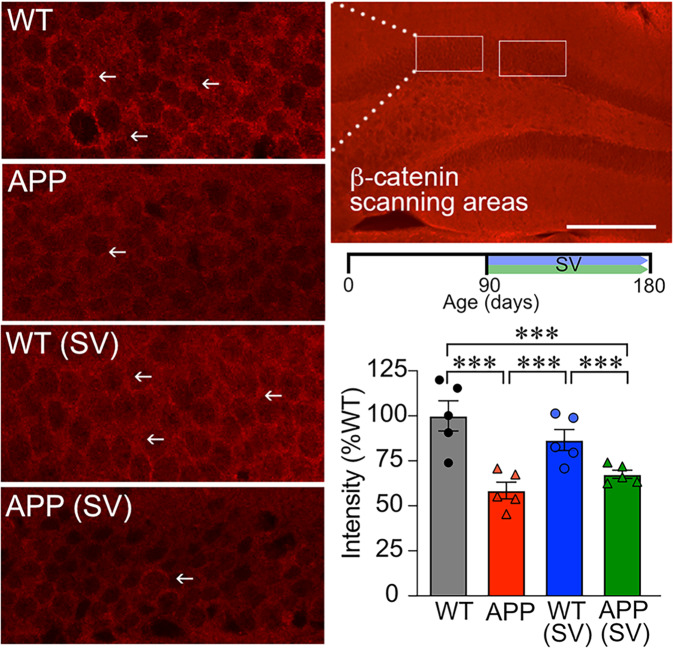
SV did not affect β-catenin in the dentate gyrus granule cell layer. β-catenin immunofluorescence staining in the mouse hippocampus was quantified in the granule cell layer (white boxes in right top panel) shown on the confocal images (left panels). Fine β-catenin-immunostained punctate structures were localized in the neuropil surrounding the granule cells in WT controls (arrows, left panels); structures that were significantly less intensely labeled in APP mice. SV treatment (timeline shown on top of bar graph) had no effect as analyzed by MetaMorph software (bottom at right panel). ***p < 0.001, using two-way ANOVA followed by Newman–Keuls post-hoc test. Bar = 200 µm.

**Fig. 7:**
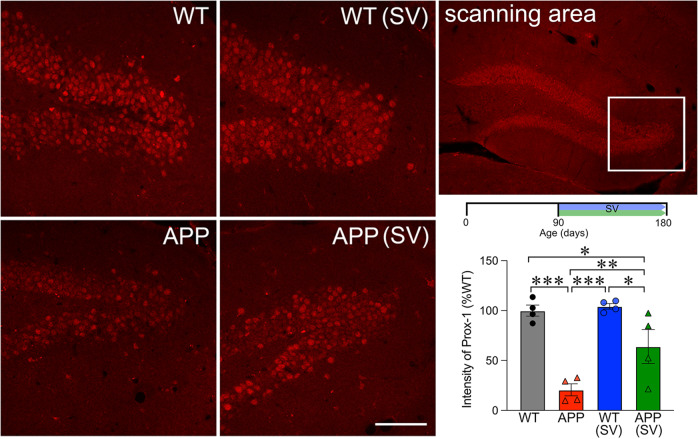
SV increased Prox1 expression in granule cells of APP mice. The white box in the top right panel shows the confocal scanning area for Prox1 immunofluorescence staining in the dentate gyrus granule cell layer. Prox1 protein levels were drastically reduced in granule cells from APP mice compared to WT controls. Although SV treatment (timeline shown on top of bar graph) significantly increased Prox1 levels in treated APP mice, they still remained significantly lower than those of WT mice. (*n* = 4–5 mice/group) ***p < 0.001, using two-way ANOVA followed by Newman-Keuls post-hoc test. Scale bar = 75 µm.

When investigating a potential role for SV on the Wnt/β-catenin pathway, we first analyzed β-catenin immunopositive material in the granule cell layer of the DG on confocal images. In WT mice, dotted β-catenin-immunopositive material surrounded the perikarya of CA1 granule cells (Fig. 6). This labeling was reduced (↓40%, *p* < 0.001) in APP mice, and not significantly increased in APPSV mice (↑10%, ns). We then examined Prox1 (Prospero-related homeobox 1 gene), a β-catenin-TCF/LEF signaling targeting protein involved in hippocampal neurogenesis [33]. Prox1 was found in the cytoplasm of granule cells, and its levels were significantly lower in APP mice compared to WT controls (Fig. 7, ↓77%, *p* < 0.001). SV significantly increased Prox1 protein levels in treated APP mice (↑ 63%, *p* < 0.01), although they were still reduced compared to WT. These results suggest that reduced Prox1 expression in APP mice likely results from altered signaling in the Wnt/β-catenin pathway, possibly contributing to the impaired neurogenesis such as defective granule cell maturation.

### SV enhances neurogenesis and improves memory through the Wnt/β-catenin pathway

**Fig. 8:**
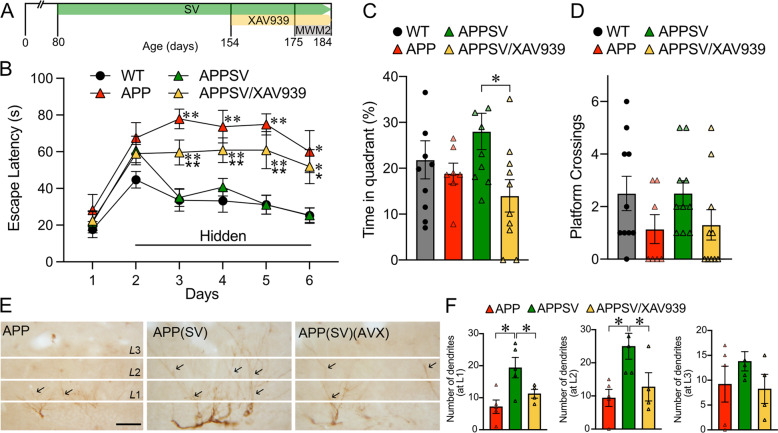
Wnt signaling mediates SV beneficits on memory and dentritic extensions. **A** Schematic representation of experimental design and timeline of SV and the Wnt/β-catenin signaling inhibitor XAV939 treatment. **B** APP mice (red triangular) displayed impaired spatial learning during the hidden-platform testing in the Morris water maze (MWM2) compared to WT (black circle), a deficit prevented by SV treatment (APPSV mice, green triangle) and totally abrogated in APP mice that received the Wnt/β-catenin signaling inhibitor XAV939 during the last month of SV treatment (APPSV/XAV939, yellow triangles). These deficits were not due to visual or motor disabilities as APPSV/XAV939 mice performed as well as the other groups in finding the visible platform (day 1). **C** During the probe trial, despite poorer performance in APP mice and clear improvement in APPSV mice, significance was not reached. However, SV benefits were completely lost in APPSV/XAV939 mice as shown here for the time spent in the target quadrant (**C** *p* < 0.05) and crossings over the previous location of the hidden platform (**D** not significant). **E** Representative images of DCX immunopositive dendrites (black arrows) in the DG molecular layer were divided into L1, L2, and L3 segments. **F** Quantitative analysis showed that SV increased dendritic length in L1 and L2 segments, benefits lost in APPSV mice that received the Wnt signaling inhibitor XAV939 during the last month of SV treatment. *APP and SV**/**XAV939-treated APP mice compared to WT; APPSV/XAV939 mice compared to APPSV mice; *p < 0.05; **p < 0.01 using one-way ANOVA and repeated measures ANOVA followed by Newman-Keuls post-hoc test. Scale Bar = 50 µm.

To further decipher the contribution of the Wnt/β-catenin pathway in the beneficial effects of SV memory function and granule cell maturation, we used icv administration of the selective Wnt/β-catenin pathway inhibitor XAV939 (Fig. 8A). All groups of APP and WT mice had no difficulty finding the visible platform (day 1, Fig. 8B). In the spatial learning component of MWM2, APP mice showed longer time latencies compared to WT, an impairment fully countered in APPSV mice as shown by their similar performance to WT. APP mice concomitantly treated with SV and the Wnt/β-catenin pathway inhibitor XAV939 performed as poorly as untreated APP mice indicating that SV’s benefit was lost when blocking the Wnt/β-catenin pathway (Fig. 8B). In the probe trial, the time spent in the target quadrant was significantly reduced in XAV939-treated APPSV mice compared to APPSV treated mice (Fig. 8C). All other parameters measured in the probe trial showed the expected deficits in APP mice, benefits of SV and worsening effects of XAV939, but these did not reach significance, as shown here for the platform crossings (Fig. 8D). Moreover, XAV939 blocked SV-mediated benefits on DCX-dendritic extension in the molecular layer of the DG (Fig. 8E), reducing significantly the number of dendrites in the L1 and L2 segments of the molecular layer to that of untreated APP mice (Fig. 8F). These results demonstrate that blocking Wnt/β-catenin signaling eliminated SV benefits on spatial memory and granule cell maturation.

## Discussion

Together the findings of the present study establish a key role for the Wnt/β-catenin pathway in the cognitive and neuronal benefits of SV in APP mice leading to improved functionality within the “corticohippocampal memory circuit”. Our results show that (1) SV-treated APP mice recover memory concurrently with activation of the Wnt/β-catenin pathway and improved maturation and dendritic extension of DG granule cells, (2) selective blockade of Wnt/ β-catenin signaling counters SV benefits, and (3) SV’s memory benefits were replicated by selectively activating the Wnt/β-catenin pathway via inhibition of DKK1, an endogenous inhibitor of the Wnt/β-catenin pathway upregulated in brain tissue of both APP mice [32] and AD patients [31].

### Neuronal maturation in the DG and SV treatment

Our findings of increased proliferating Ki67 cells in young APP mice followed by a decrease in both proliferating and immature (DCX-immunopositive) neurons at 4 months of age that persisted until 6 months, agree with previous reports of age-related increased or decreased neurogenesis [34–37]. These observations, together with the reduced number of mature granule cells in adult (6 months-old) APP mice, as previously reported [38], point to impaired differentiation and maturation of DG newborn neurons into functional mature granule cells [39]. Further, the reduced length of the dendritic arborizations of immature (DCX) and mature (calbindin) granule cells observed here recapitulates the abnormal dendritic development reported in Tg2576, APPxPS1, and APPSw,Ind mice that was attributed to soluble Aβ species [36, 38, 40]. Immature and mature granule cells with shorter dendritic extensions will likely be unable to fully capture inputs from the performant path; hence, impairing their integration into a functional corticohippocampal memory network underlying spatial memory performance in mice [36].

Interestingly, 90 days of SV therapy, irrespective of the age of treatment initiation (30 to 90 days old mice) did not increase the number of proliferating (Ki67) or immature (DCX) neurons in the DG, but fully normalized the dendritic length of DCX neurons measured in 6 month-old adult APP mice. What is more, when looking at mature (calbindin) granule cells in 6 month-old APPSV mice with restored memory, despite no significant increases in their number and CA3 axonal afferents, we found a full recovery in the length of their dendritic arborizations in the molecular layer of the DG. Whether dendritic spine density would also be rescued by SV, as observed for atorvastatin in diabetic rats [41], requires further investigation. Similar findings of improved or fully restored hippocampal-dependent cognitive tasks and dendritic length of DCX neurons were reported in APPSw, Ind mice after 7 weeks of enriched environment [38]. Moreover, cognitive rescue and normalized dendritic spine density was observed in APPxPS1 mice with only a small population of granule cells with genetically accelerated maturation being integrated into functional memory pathway [40]. These results align well with our findings of persevering reduced DCX neurons in the granule cell layer in APPSV treated mice that displayed full memory recovery. These observations further argue that a small number of granule cells functionally integrated in the memory pathway is sufficient for counteracting memory deficits in APP mice [40]. Overall, our and previous findings in various pre-clinical AD mouse models agree with the reported impaired neurogenesis in AD patients characterized by newly generated neurons not reaching maturity [42, 43]. Furthermore, our findings are supported by previously reported reduced levels of calbindin in AD brains [44, 45], alterations associated with memory deficits and suggested to be amenable to therapy [43].

### SV treatment and Wnt signaling pathway

In this respect, the Wnt/β-catenin signaling pathway [46] has been involved in DG granule cell differentiation and maturation, dendritic branching, depolarization-induced dendritogenesis, and synapse stability and plasticity [47–50]. The Wnt signaling pathway is affected in AD, with diminished brain levels of β-catenin [44, 45] and upregulation of DKK1 [31], a negative modulator of the canonical Wnt/β-catenin signaling pathway [45]. Activation of the Wnt signaling pathway has been considered of potential value in the treatment of neurodegenerative diseases that affect synaptic integrity and connectivity, including AD [49–51]. Similarly, lithium, through facilitation of β-catenin nuclear translocation and activation of Wnt/β-catenin target genes, was found to promote neuronal differentiation and improve cognitive impairments in young (3 months-old) TgCRND8 mice [52]. Most interesting is the fact that SV, acting through Wnt signaling, has been shown to enhance neurogenesis in cultured adult neural progenitor cells as well as in the DG of adult mice [26]. Here, we find that SV not only rescued memory in adult APP mice, but significantly increased the immunopositive protein levels of Prox1 in the APP mouse DG granule cell layer, Prox1 being a Wnt/β-catenin target gene required for initial granule cell differentiation and maturation [33]. This Prox1 upregulation likely contributes, together with other factors, to the improved dendritic length of both immature (DCX) and mature (calbindin) granule cells within the DG molecular layer seen in APPSV mice. In this respect, hippocampal dendritogenesis, particularly dendritic arborization, is facilitated by brain-derived growth factor (BDNF) [53, 54], which is upregulated in other AD mouse models treated with SV [16, 55].

Additionally, we found barely detectable levels of immunopositive DKK1 neurites in the hippocampus of WT mice, levels that were drastically increased in APP hippocampi as reported for DKK1 protein levels in AD brains [45], and greatly reduced in SV-treated 6 months-old APP mice. Correspondingly, APP mice treated with the DKKi and selective activator of the β-catenin-dependent Wnt pathway WAY-262611 showed normal performance in the MWM, increased hippocampal β-catenin protein levels, and slightly albeit not significantly, improved dendritic length of DCX neurons. These findings are consistent with previous reports of enhancing effects of WAY-262611 on β-catenin levels in aged APPswe/PS1 mice [56], and of restored memory in old DKK1 deficient mice with newborn mature neurons exhibiting a more elaborated dendritic morphology [57].

The link between SV and the Wnt/β-catenin signaling pathway in rescuing memory and DG granule cell maturation in APP mice was further substantiated in APPSV mice that received the WnT/β-catenin signaling inhibitor XAV939 during the last month of SV treatment. Remarkably, the XAV939 treatment abolished SV’s benefits on both memory and dendritic morphology. Taken together with the findings obtained with the DKKi WAY-262611 and selective activator of the Wnt/β-catenin pathway, our results unequivocally demonstrate that SV acts, at least in part, through activation of the canonical Wnt/β-catenin pathway to induce its memory and neuronal benefits. Interestingly, DKK1 was recently recognized as a target for statin benefits on endothelial cells [58]. It is thus possible that the ability of SV to rescue cerebrovascular function and, particularly, endothelial-dependent dilations in adult and aged APP mice [14, 15] also relates to counteracting defective Wnt signaling.

Yet, crosstalk with other pathways cannot be excluded. Indeed, SV protective benefits on neurogenesis in Aβ25-35-injected mice [16], as well as on synaptic plasticity and long-term potentiation in APPSwe/PS1dE9 mice [21] have been associated with activation of GSK-3/AKT and ERK/AKT signaling pathways, both known to regulate Wnt/β-catenin signaling [59, 60]. Moreover, DKK1 promoted degeneration of hippocampal functional circuits by blocking canonical Wnt/β-catenin signaling and activating the RhoARock pathway [49], a pathway inhibited by SV [61] and which inhibition resulted in improved memory [62]. Statins also modulate neurogenesis through inhibition of the mevalonate pathway by suppressing isoprenylation and geranylgeranylation of small GTPases such as RhoA [26, 63–65], which could result in upregulation of Wnt signaling [65, 66], possibly via blockade of DKK1 as in other pathologies with elevated DKK1 [67]. Other protective mechanisms may also contribute to SV’s benefits. For instance, statins attenuate mitochondrial activity and the endoplasmic reticulum unfolded protein response (UPR) in APP/PS1 mice [68] or following an acute stroke [69], which reportedly enhance autophagy in models of brain injury [70–72]. Wnt-mediated modulation of autophagy has also been associated with beneficial effects in models of brain injury and dementia [73–76]. Yet, a role for autophagy or the UPR in the cognitive and structural benefits of SV observed here in adult APP mice requires further investigation. SV’s potential for improving the clinical outcome of AD patients is further supported by its ability to improve attentional performance together with decreased hippocampus neuronal damage in 3xTgAPP mice by modulating expression of anti- and pro-apoptotic genes [13], notwithstanding its anti-inflammatory, antioxidant and antiapoptotic effects [14, 77], as well as ability to improve the survival rate of neurons [78].

## Conclusions

Our results demonstrate that the Wnt/β-catenin pathway underlies the cognitive and neuronal benefits instigated by SV in APP mice and identify a potential new therapeutic target in AD. Our results highlight the importance of ongoing efforts aimed at developing or improving new brain penetrant statin derivatives with improved efficacy and lessened negative effects [79], or DKK1 antagonists [57, 80] to promote or protect neurogenesis, neuronal connectivity and cognitive function in AD patients. Alternative strategies could aim at preventing DKK1 binding to the Wnt co-receptor LRP6 as suggested in other neurodegenerative diseases [81]. It also appears that targeting DKK1 may contribute to rescue of AD-related cerebrovascular dysfunction [56, 58].

## Materials and methods

### Animals

Heterozygous transgenic C57BL/6 mice that express the amyloid precursor protein (APP) carrying the human Swedish (K670N, M671L; APPSwe) and Indiana (V717F; APPInd) familial AD mutations directed by the PDGF β-chain promoter (APP mice, J20 line) [82] and wild-type (WT) mice were used, with approximate equal numbers of males and females. APP J20 mice display early (2–4 months) cerebrovascular deficits [83], increased levels of soluble Aβ species [82] that precede the formation (5–6 months) of diffuse [82] and dense-core Aβ plaques [84], and develop progressive cognitive deficits fully manifest by 4–6 months of age [15, 44, 85]. Experiments abided to the Animal Ethics Committee of the Montreal Neurological Institute (McGill University, Montréal, QC, Canada) and complied with the regulations of the Canadian Council on Animal Care and the ARRIVE guidelines.

### Treatments

#### Simvastatin (SV)

Simvastatin (Enzo Life Sciences International) was activated by alkaline lysis according to the manufacturer’s protocol, and added to the drinking water such that mice received ∼40 mg/kg of body weight/day, a dose similar to or lower than that used in previous studies looking at central effects of SV [14, 15, 20]. SV was administered at 20 mg/kg/day for 3 days, increased to 30 mg/kg/day for 4 days, and then to 40 mg/kg/day for the remainder of the treatment [15]. Controls received the same drinking solution without SV.

#### WAY-262611 and XAV939

Mice were surgically implanted with subcutaneous osmotic mini-pumps (2.64 µl delivery/day; Alzet, Cupertino, CA) connected to an intracerebroventricular (icv) catheter positioned within the left cerebral ventricle (AP: Bregma −0.46 mm; L: 1 mm.according to Atlas of mice) [86]. Pumps delivered either the DKKi WAY-262611 (a selective activator of the Wnt/β-catenin pathway acting through inhibition of DKK1, 10 μg/day, Enzo life science, Famingdale, NY, USA), the Wnt/β-catenin signaling inhibitor XAV939 (0.5 nmol/hr, Enzo life science), or vehicle (artificial CSF, 1:1 solutions A and B, solution A (500 ml): NaCl 8.66 g, KCl 0.224 g, CaCl2 0.206 g and MgCl2 0.163 g; solution B (500 ml), Na2HPO4 0.214 g and NaH2PO4 0.027 g) for 28 days; doses were adjusted or selected from their reported in vivo effects on peripheral [87] or centrally-mediated [88] pathways.

#### Experimental design

The following cohorts of mice were used: (1) *the developmental cohorts*: 10 groups of WT and APP mice (*n* = 5–10/group) aged 20, 30, 40, 60, 90, 120, 135, 150, 165, and 180 days; (2) *the simvastatin (SV)-treated cohorts*: Mice received 3 months (90 days) of SV, treatment was initiated at 30, 45, 60, 75, and 90 days of age, with respective endpoints at 120, 135, 150, 165, and 180 days of age. For each age point, mice were divided into four groups (*n* = 4–7/group): WT, WT mice treated with SV (WTSV), APP and APP mice treated with SV (APPSV); (3) *the cohorts treated with the Wnt/β-catenin agonist or Dickkopf-related protein 1 (DKK1) inhibitor (DKKi) WAY-262611*: Two independent cohorts of WT and APP mice treated with or without WAY-262611 (*n* = 12–14/group); and (4) *the cohorts treated with the Wnt/β-catenin signaling inhibitor XAV939*: Two cohorts consisting of WT (vehicle), APP, APPSV, and APPSV mice treated concurrently with XAV939 for the last month of SV treatment (*n* = 7–10/group). The sample size for each experiment was based on our previous studies. Mice were randomly distributed between the different treatment groups based on sex and equivalent performance in the Morris water maze 1 (MWM1, see below). The experimentator was blinded to the identify and treatment of the mice, and all mice were included in the analysis.

### Morris water maze

#### Morris water maze (MWM)

Spatial learning and memory were tested in a modified version of the MWM, as previously described [15, 85]. For cohorts undergoing only one MWM testing (MWM 1), the paradigm consisted of 8 days of two training sessions in a circular pool (1.4 m diameter, 0.4 m deep) filled with opaque water (18 ± 1 °C, containing non-toxic tempera painting powder, Discount, School Supply) located in a quiet room with distal visual cues. Animals were first familiarized with the test for 3 days by searching a visible platform (days 1–3, 60 s/trial), followed by a 5-day training session (days 4–8, 3 trials/day, 90 s/trial max) whereby mice had to learn the location of a hidden platform (∼1 cm below the surface of the water). Platform and visual cue location were changed between the two training sessions. A 45 min inter-trial interval was respected. Spatial memory was evaluated during the probe trial (platform removed) performed on day 9 (60 s/trial, 1 trial). Escape latencies and probe trial parameters (percent time spent and distance traveled in the target quadrant where the platform used to be located, number of crossings over the previously located platform, and swim speed) were recorded with the 2020 Plus tracking system and Water 2020 software (Ganz FC62D video camera; HVS Image, Buckingham, UK). Mice were kept warm with a heating lamp to avoid hypothermia. Subsequent experiments started 2 days later.

For the DKKi-treated cohort, MWM 1 was performed on mice at 140 days of age and included WT (*n* = 13) and APP (*n* = 28) mice. Following MWM 1, mice were randomly divided into two groups: one group received icv infusion of WAY-262611 and the other received vehicle. A second maze (MWM 2) was initiated when the mice were 171 days old, and was completed at 180 days of age with mice receiving DKKi treatment for 30 days. For MWM 2, only 1 day of familiarization was used and the location of the visual cues, visible and hidden platforms differed from MWM1.

For the Wnt/β-catenin signaling inhibitor XAV939-treated mice, SV treatment was initiated in 80 day-old mice and continued for 2 months until MWM 1 testing at 145 days of age. After MWM 1, mice (then 154 days old) were implanted with osmotic minipumps and icv catheters and randomly divided into groups for concurrent delivery of XAV939 or vehicle for the last month of SV treatment. MWM 2 was performed in mice from 175 to 184 days of age.

### Tissue preparations

After completing MWM experiments, some mice (*n* = 4–5 mice/group) were decapitated and hippocampi extracted, frozen on dry ice and stored (−80 °C) for protein extraction for Western blot analysis. Another subset of mice (*n* = 4–5 mice/group) were perfused intracardially with 20 ml cold saline followed by 200 mL of 4% paraformaldehyde (PFA) in cold 0.1 M phosphate buffer (PB, pH 7.4, 4 °C), their brains were post-fixed in 4% PFA (overnight, 4 °C) and transferred to 30% sucrose (48 h, 4 °C) for cryoprotection until freezing in isopentane (−40 °C). The brains were then stored (−80 °C) until sectioning (25 µm thickness) on a freezing microtome for anatomical studies.

### Western blotting

Hippocampal proteins (∼20 µg, 4–5 mice/group) were extracted and assayed (BioRad), separated using a 10% SDS PAGE and transferred to nitrocellulose membranes for the detection of β-catenin protein levels (rabbit anti-β-catenin, Santa Cruz, California, USA). Membranes were subsequently incubated (1 h) with anti-rabbit horseradish peroxidase-conjugated secondary antibodies (1:2000; Jackson ImmunoResearch, West Grove, PA, USA) in TBST blocking buffer (50 mM Tris-HCl, pH = 7.5; 150 mM NaCl; 0.1% Tween 20) containing 5% skim milk, and visualized with enhanced chemiluminescence (ECL Plus kit; Amersham, Baie d’Urfé, QC, Canada) using a phosphorImager (Scanner STORM 860; GE Healthcare, Baie d’Urfé, QC, Canada). Band intensity was quantified by densitometry with Scion Image (Molecular Dynamics, Sunnyvale, CA, USA).

### Immunostaining

Free-floating sections were incubated (overnight, room temperature) with rabbit anti-Ki67 (1:1000, Leica, Cedarlane, Burlington, ON, Canada) or goat anti-doublecortin (DCX, 1:1000, Santa Cruz), then incubated in biotinylated species-specific secondary antibodies and the Avidin-Biotin complex (ABC kit, Vector laboratory). The immunoreactive material was detected in 3′-diaminobenzidine (DAB, brown precipitate). Immunoflurorescence staining with rabbit anti-calbindin (1:10000, Swant, Switzerland), anti-β-catenin (1:100, Santa Cruz), anti-Prox1 (1:3000, Millipore, Temecula, CA), or goat anti-DKK-1 (1:60, R&D system, Minneapolis) was detected with species-specific cyanin-3 (Cy3) or Alexa 594 (red) conjugated secondary antibody (Jackson Labs, West Grove, PA, USA). Sections (minimum 2-3/mouse) were observed under light microscopy or epifluorescence on a Leitz Aristoplan microscope (Leica, Montréal, QC, Canada), or confocal microscopy (LSM 510 or 710, Zeiss), digital pictures were taken and used for analysis. Staining specificity was confirmed by omitting primary antibodies.

### Statistical analysis

Low and high magnification digital pictures were used to count the number of Ki67- or DCX-immunopositive nuclei or neurons in the granule cell layer of the dentate gyrus (DG), and measure the number of DCX dendrites and the length of their projections in the DG molecular layer. For some analyses, the molecular layer was divided in three segments (L1, L2, and L3, L1 being the most proximal to the DCX cell bodies and L3 the most distal) to better capture the level of changes. Image J (NIH, Bethesda, MD, USA) and MetaMorph 6.1r3 (Universal Imaging, Downington, PA, USA) were used to quantify the intensity of mossy fibers and calbindin-immunostained fibers in the DG molecular layer. We used high magnification confocal images and semi-quantitative analyses to measure β-catenin-, Prox1-, and DKK-1-immunopositive material. For the latter, since no or virtually no DKK-1-immunostaining was detected in WT mice, semi-quantitative measures were compared in APP and APPSV mice. Data were expressed as mean ± SEM and analyzed by Student’s *t* test, one-way or two-way (genotype and treatment as factors) ANOVA followed by Newman–Keuls *post hoc* multiple comparison test (GraphPad Prism4, San Diego, CA, USA). *P* < 0.05 was considered significant.

## Supplementary information

Supplementary Material

Reproducibility checklist
