# Supplementary material for: Simvastatin rescues memory and granule cell maturation through the Wnt/β-catenin signaling pathway in a mouse model of Alzheimer’s disease
Source: Cell Death Dis. 2022 Apr 9;13(4):325. doi: 10.1038/s41419-022-04784-y (PMC8994768; doi:10.1038/s41419-022-04784-y)
Supplement: Supplementary file 1 — Supplementary Material [file 41419_2022_4784_MOESM1_ESM.docx]

**Supplementary Material**

Simvastatin rescues memory and granule cell maturation through the Wnt/β-catenin signaling pathway in a mouse model of Alzheimer’s disease

Tong Xin-Kang, Royea Jessika^1^, and Hamel Edith

Laboratory of Cerebrovascular Research, Montreal Neurological Institute, McGill University

3801 University Street, Montréal, QC, Canada H3A 2B4

Running title: Simvastatin, memory and the Wnt pathway


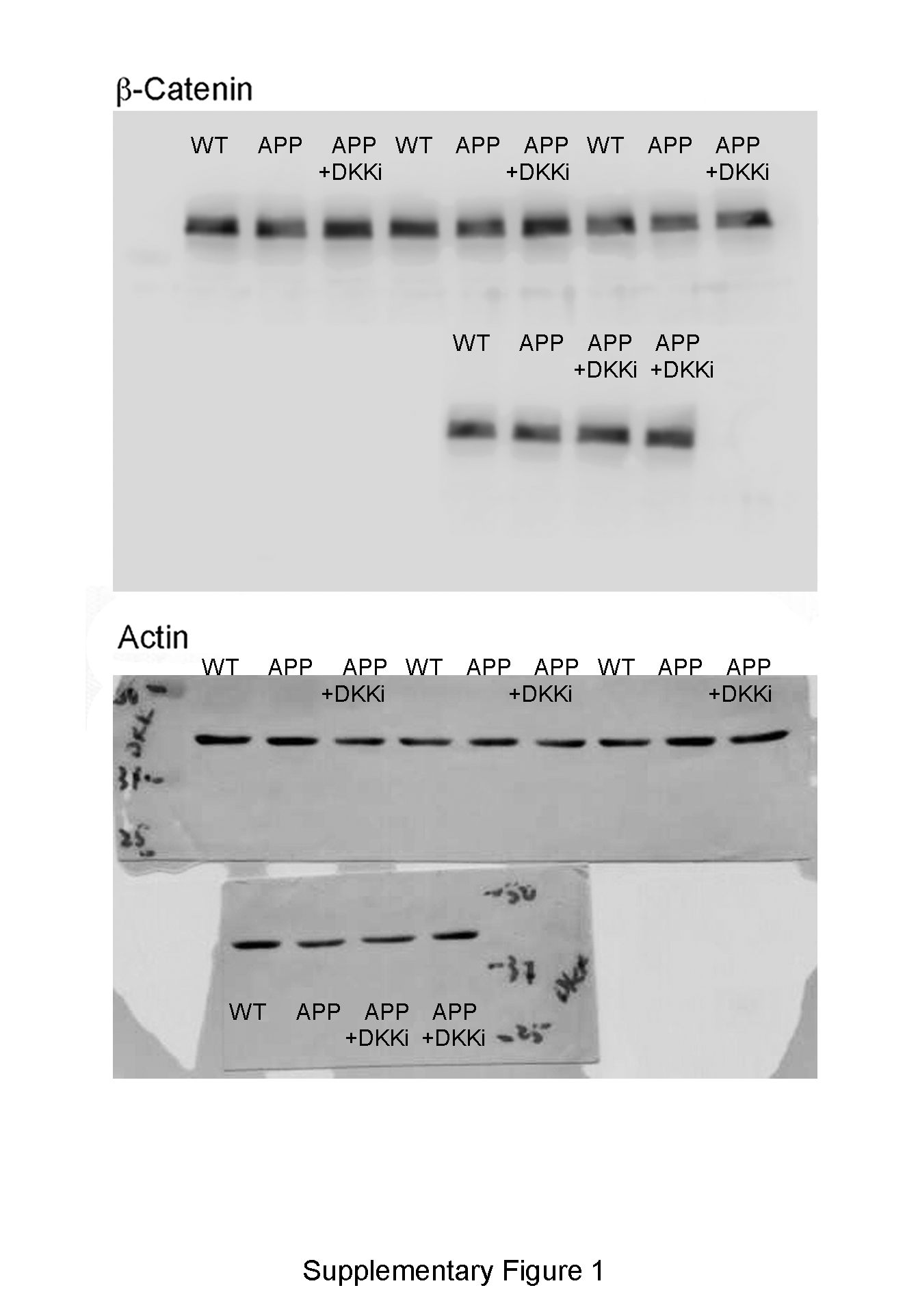


**Supplementary Figure 1**: Original Western blots for β-catenin and actin protein levels in hippocampus from wild-type (WT) and APP mice treated (APP+DKKi) or not (APP) with the selective activator of the Wnt/β-catenin pathway and DKK1 inhibitor (DKKi) WAY-262611. Quantitative analysis showed no significant difference between groups.
